# Cytokinin deficiency confers enhanced tolerance to mild, but decreased tolerance to severe salinity stress in *in vitro* grown potato

**DOI:** 10.3389/fpls.2023.1296520

**Published:** 2024-02-01

**Authors:** Martin Raspor, Miloš Mrvaljević, Jelena Savić, Tatjana Ćosić, Abdul Rasheed Kaleri, Nina Pokimica, Aleksandar Cingel, Nabil Ghalawnji, Václav Motyka, Slavica Ninković

**Affiliations:** ^1^ Department of Plant Physiology, Institute for Biological Research “Siniša Stanković” - National Institute of Republic of Serbia, University of Belgrade, Belgrade, Serbia; ^2^ School of Life Science and Engineering, Southwest University of Science and Technology, Mianyang, China; ^3^ Laboratory of Hormonal Regulations in Plants, Institute of Experimental Botany of the Czech Academy of Sciences, Prague, Czechia

**Keywords:** antioxidant enzymes, chlorophyll, cytokinin oxidase/dehydrogenase, *in vitro*, potato, salinity, transgenic, water saturation deficit

## Abstract

Cytokinin (CK) is a plant hormone that plays crucial roles in regulating plant growth and development. CK-deficient plants are widely used as model systems for investigating the numerous physiological roles of CK. Since it was previously shown that transgenic or mutant CK-deficient *Arabidopsis* and *Centaurium* plants show superior tolerance to salinity, we examined the tolerance of three CK-deficient potato lines overexpressing the *Arabidopsis thaliana CYTOKININ OXIDASE/DEHYDROGENASE2* (*AtCKX2*) gene to 50 mM, 100 mM, 150 mM, and 200 mM NaCl applied *in vitro*. Quantification of visible salinity injury, rooting and acclimatization efficiency, shoot growth, water saturation deficit, and chlorophyll content confirmed that the CK-deficient potato plants were more tolerant to low (50 mM) and moderate (100 mM) NaCl concentrations, but exhibited increased sensitivity to severe salinity stress (150 and 200 mM NaCl) compared to non-transformed control plants. These findings were corroborated by the data distribution patterns according to principal component analysis. Quantification of the activity of superoxide dismutases, peroxidases, and catalases revealed an impaired ability of *AtCKX2*-transgenic lines to upregulate the activity of antioxidant enzymes in response to salinity, which might contribute to the enhanced sensitivity of these potato lines to severe salt stress. Our results add complexity to the existing knowledge on the regulation of salinity tolerance by CK, as we show for the first time that CK-deficient plants can exhibit reduced rather than increased tolerance to severe salt stress.

## 1 Introduction

A major point of concern for the world’s sustainable development is food security, due to the rapid increase in global human population on the one hand, and extensive land degradation and climate change on the other (Liu et al., 2020; Tan et al., 2022; Teo et al., 2022). Potato (*Solanum tuberosum* L.) is the world’s fourth most important food crop, after maize, wheat, and rice. It is a staple crop in developing countries and is considered a crop of crucial importance for global food security (Ahmadu et al., 2021).

In addition to tuber yield, salt tolerance is becoming a topic of increasing importance for potato biology, due to progressive salinization of agricultural lands in developing countries (Chourasia et al., 2021). Soil salinization affects plant growth and harms their survival and yield through both osmotic stress and the toxic effects of Na^+^ and Cl^-^ ions (Munns and Tester, 2008; Flowers et al., 2015). The cytotoxic effects of Na^+^ ions include interference with cellular K^+^ uptake, cytosolic accumulation of Na^+^, and the resulting damage to housekeeping enzymes (Pardo and Quintero, 2002; Kronzucker and Britto, 2011). At the whole-plant level, the toxic effects of salinity on root cells lead to root growth arrest (Verslues et al., 2006; Yildiz et al., 2021). In photosynthetic tissues, high concentrations of Na^+^ and Cl^-^ disrupt electron transport and cause physical damage to thylakoid membranes, resulting in the leakage and degradation of chlorophyll (Muhammad et al., 2021). The reduction of photosynthetic efficiency, along with damage to meristematic tissues, results in shoot growth arrest (Munns and Tester, 2008; Muhammad et al., 2021). In addition to toxic effects specific to Na^+^ and Cl^-^ ions, salinity causes osmotic stress that further impairs nutrient mobility and photosynthesis (Hanin et al., 2016). The multiple toxic effects of salinity on plant tissues lead to the accumulation of reactive oxygen species, causing oxidative stress, which additionally contributes to cell damage and death (Munns and Tester, 2008).

Plants have evolved a variety of adaptations that allow them to tolerate salinity to various extents. These mechanisms include: stomatal closure for the reduction of water loss through transpiration (Jung et al., 2008; Campos et al., 2016); selective transport and compartmentalization of ions (Pardo and Quintero, 2002; Kronzucker and Britto, 2011); production of protective osmolytes (Sharma et al., 2019); and enzymatic and non-enzymatic mechanisms of protection from oxidative stress (Ahmad et al., 2010). Plant species are broadly divided into halophytes (tolerant to salinity) and glycophytes (sensitive to salinity); however, the efficiency of particular tolerance mechanisms can greatly vary even between different genotypes of the same species (Munns and Tester, 2008; Flowers et al., 2015). Like most economically important crops, potato is considered moderately sensitive to salinity, which makes the physiological regulation of its tolerance an important topic for research (MaChado and Serralheiro, 2017; Chourasia et al., 2021).

To adequately address the challenges associated with food security, global research in agricultural botany focuses on plant hormones as key regulators of physiological processes related to agronomically important outputs such as yield or stress tolerance (Ciura and Kruk, 2018). Among plant hormones, cytokinin (CK) holds a central place within the regulatory networks that control plant growth and development. Although tolerance to abiotic stress is mainly regulated by abscisic acid (ABA), salicylic acid (SA) and jasmonic acid (JA), important involvement of CK in the regulation of responses to abiotic stress, particularly salinity, has gained scientific attention within the last decade (Mýtinová et al., 2010; Nishiyama et al., 2011; Nishiyama et al., 2012; Wu et al., 2012; Liu et al., 2013; Joshi et al., 2018; Li et al., 2019; Trifunović-Momčilov et al., 2020; Zhu et al., 2020; Abdelrahman et al., 2021; Trifunović-Momčilov et al., 2021).

In potato, the physiological roles of CK have been studied mainly in the context of tuber development (Romanov et al., 2000; Kolachevskaya et al., 2021), including transgenic overexpression of genes encoding the CK catabolic enzyme cytokinin oxidase/dehydrogenase (CKX; EC 1.4.3.18/1.5.99.12), providing insight into the complex effects of CK deficiency on potato tuber development (Hartmann et al., 2011; Raspor et al., 2012; Raspor et al., 2021). Since CK deficiency has already been reported to positively affect the salt tolerance of plants such as *Arabidopsis thaliana* (Nishiyama et al., 2011; Li et al., 2019) and *Centaurium erythraea* (Trifunović-Momčilov et al., 2020), in this work we aimed to investigate whether CK deficiency could confer increased salt tolerance to the *AtCKX*-transgenic potato plants. The *AtCKX2*-transgenic lines were used in this study because their tuber size and yield were less negatively affected by CK deficiency compared to the *AtCKX1* lines (Raspor et al., 2021).

## 2 Materials and methods

### 2.1 Plant material

Potato (*Solanum tuberosum* L. cv. Désireé) lines overexpressing the *Arabidopsis thaliana CYTOKININ OXIDASE/DEHYDROGENASE2* gene (*AtCKX2*) were obtained as previously described (Raspor et al., 2012). The *AtCKX2*-overexpressing lines *AtCKX2*-39, *AtCKX2*-48 and *AtCKX2*-51 were selected based on the highest levels of transgene expression as evidenced in our previous work (Raspor et al., 2012). In addition, a wild-type potato line was used as a non-transformed control. The potato lines were micropropagated on MS nutrient media supplemented with vitamins (Murashige and Skoog, 1962; Linsmaier and Skoog, 1965), 3% sucrose, and 0.7% agar. Five plantlets of each genotype were grown together in 200 mL glass jars (6-10 jars per genotype and treatment for each measurement). The jars were placed in a growth chamber with white fluorescent light (Philips TL-D 58 W/54-765, 58 W, 6200 K, 50 ± 3 µmol m^-2^ s^-1^) at a 16/8 h photoperiod and a temperature of 23 ± 2°C. The experiments were repeated three times with similar results.

### 2.2 Salinity treatments and morphometric measurements

For salinity treatments, single-node stem cuttings (SNCs) of 20-day-old *in vitro* grown plants were cultured on MS media supplemented with 0, 50, 100, 150, or 200 mM NaCl. Plants regenerated from the axillary meristems of SNCs were sampled after 20 days of salt treatment for morphometric measurements, determination of chlorophyll content, and the activity of antioxidant enzymes, as described below.

#### 2.2.1 Salinity injury index

The salinity injury index (SII) (Diaz-Vivancos et al., 2013; Savić et al., 2019) was evaluated as a general measure of salt-induced damage to the stressed plants, as described in **Table 1**. Modifications to the previous descriptions of SII (Diaz-Vivancos et al., 2013; Savić et al., 2019) were introduced to match the specific effects of salinity damage to the *in vitro* grown potato plants. The SII values for a particular genotype at a given experimental treatment were calculated as the mean of the individual scoring of at least 30 plantlets, performed independently by two researchers with almost identical results.

**Table 1:** Criteria for scoring the salinity injury index (SII) of *in vitro* grown potato.

Salinity Injury Index	General characteristics
0	Normal appearance; green shoot and dark green leaves; normally developed root system
1	The shoot, root, and leaves look almost normal, but shoot and leaves are distinctly lighter in color; both shoot and root are slightly to moderately stunted
2	The shoot is severely stunted and yellowish in color, with very short internodes and brittle, yellow leaves; the root system is severely stunted or absent
3	The axillary shoot is necrotic or not developed at all; no root system

Modified from: Diaz-Vivancos et al. (2013); Savić et al. (2019).

#### 2.2.2 Rooting efficiency

Rooting efficiency was calculated for a particular genotype in a given experimental treatment as the proportion of rooted plantlets among at least 30 plantlets. Mean rooting efficiency was calculated as the average of three independent experiments.

#### 2.2.3 Acclimatization assays

After 20 days of exposure to salinity, rooted plantlets were selected from the 0, 50, 100, and 150 mM NaCl treatments and transplanted into pots containing a 3:1 potting soil/vermiculite mixture, grown in a greenhouse at 25 ± 4°C, and regularly watered. At least 30 plantlets of each genotype were used from the 0 and 50 mM NaCl treatments, at least 20 plantlets of each genotype were used from the 100 mM NaCl treatment, and 5-10 plantlets of the control, *AtCKX2*-39, and *AtCKX2*-48 plantlets were used from the 150 mM NaCl treatment due to limited availability of rooted plant material. The survival rate of plants grown in soil was measured after 7 days. The experiment was repeated three times with similar results.

#### 2.2.4 Relative plant height and relative shoot fresh weight

Plant height and shoot fresh weight were calculated as mean values of at least 30 plantlets. For each genotype, plant height and shoot fresh weight at the different salinity levels were calculated relative to the mean value of plant height, or shoot fresh weight, respectively, of the same genotype at 0 mM NaCl, for example:


Relative plant height (50 mM NaCl) = Plant height (50 mM NaCl) / Mean plant height (0 mM NaCl)

#### 2.2.5 Water saturation deficit

Water saturation deficit (WSD) (Barrs, 1968) was measured as the mean values of 3 replicates with at least 10 leaf discs from 10 different plantlets for each replicate, as described below. A single fully developed leaf from each plant was selected and a leaf disc (~ 5 mm in diameter) was bored out of each leaf using a “leaf borer” - a small metal tube with sharp edges as described by Barrs and Weatherley (1962). Because of their small size, the leaf discs were grouped into clusters of 10. Fresh weight (FW) was immediately measured for each group of leaf discs, then leaf discs were soaked in Petri dishes filled with deionized water for 2 hours to attain turgid weight (TW). After TW was measured, the leaf discs were placed in an oven dryer at +80°C for 24 hours, after which their dry weight (DW) was measured (Barrs and Weatherley, 1962).

The measurement of FW, TW, and DW for each group of leaf discs allowed calculation of the water saturation deficit (WSD) values for each group, using the formula by Barrs (1968):


WSD = (TW−FW)/(TW−DW)

Similar to plant height and shoot fresh weight, WSD at the different salinity levels was expressed relative to the mean WSD value at 0 mM NaCl for each genotype, since comparison to 0 mM NaCl provided a more accurate insight into the specific effects of stress on WSD values at each of the NaCl concentrations. For instance:


Relative WSD (50 mM NaCl) = WSD (50 mM NaCl) / Mean WSD (0 mM NaCl)

### 2.3 Chlorophyll measurement

Total chlorophyll content was measured in 20-day-old plants grown *in vitro* at various NaCl concentrations using the method by Inskeep and Bloom (1985). Approximately 100 mg of leaf tissue was ground in liquid nitrogen using a pestle and mortar, extracted in 1 mL *N*,*N’*-dimethylformamide (Sigma Aldrich, Burlington, MA, USA), and centrifuged at 10,000 g for 10 min. The absorbance of the supernatant was measured spectrophotometrically at 664 nm and 647 nm, and the total chlorophyll content was calculated as follows:


$$Total\;Chl\;\left[mg\;g^{-1}FW\right]=\;17.9\;\times \;A_{647}+\;8.08\;\times \;A_{664}$$

For each genotype, the total chlorophyll content was calculated relative to the mean chlorophyll content of the same genotype at the 0 mM treatment, for instance:


Relative Chl (50 mM NaCl) = Total Chl (50 mM NaCl) / Mean total Chl (0 mM NaCl)

### 2.4 Activity of antioxidant enzymes

Total proteins were isolated from shoot tissue of 20-day-old plants grown *in vitro* at different salinity levels. Approximately 1 g of fresh shoot tissue was ground in liquid nitrogen using a pestle and mortar and extracted in a buffer containing 50 mM TRIS-HCl (pH 7.6), 10 mM EDTA (pH 8.0), 10% (v/v) glycerol, 1 mM phenylmethylsulfonyl fluoride (PMSF), 1 mM dithiothreitol (DTT), and 5% (w/w) polyvinylpyrrolidone (PVP). The extracts were then centrifuged at 13,000 g for 10 min at +4°C. The extraction procedure was repeated twice.

Total protein content was determined spectrophotometrically according to Bradford (1976). Bovine serum albumin (BSA) was used as a protein standard for the initial rough determination of the protein content in the samples. Subsequently, an aliquot of the sample with the highest protein concentration was diluted in a series of dilutions that were further used instead of BSA as protein standards for precise determination of protein content in the samples. This approach ensured better reproducibility of plant protein concentration measurements than using a series of BSA standards for quantification.

The activity of antioxidant enzymes in the protein extract samples was measured as described by Napar et al. (2022).

Briefly, for total peroxidase activity, 1 mL of the reaction mixture contained 20 mM Na acetate buffer (pH 5.0), 2 mM guaiacol, 30 mM H_2_O_2_, and 10 µL of the shoot protein extract. Spectrophotometric measurements at 470 nm were performed at 20 s intervals over 3 minutes to monitor guaiacol oxidation. The activity of total peroxidases was calculated as:


$$POD\;\left[U\;mg^{-1}protein\right]\;=\Delta A_{470}\times \epsilon_{TGC}{-}^{1}\times C_{prot}{-}^{1}$$

(ΔA_470_ = mean absorbance increase per minute; ϵ_TGC_ = 26.6 mM^-1^ cm^-1^ = molar extinction coefficient of the reaction product tetraguaiacol; C_prot_ = final concentration of the shoot protein extract)

For total catalase activity, 1 mL of the reaction mixture contained 1 mM EDTA/50 mM potassium phosphate buffer (pH 7.8), 20 mM H_2_O_2_, and 10 µL of the shoot protein extract. Spectrophotometric measurements at 240 nm were performed at 20 s intervals over 3 minutes to monitor the removal of H_2_O_2_ from the reaction mixture. The activity of total catalases was calculated as:


$$CAT\;\left[U\;mg^{-1}protein\right]\;=\;-\Delta A_{240}\times \epsilon_{H2O2}{-}^{1}\times \;C_{prot}{-}^{1}$$

(ΔA_240_ = mean absorbance decrease per minute; ϵ_H2O2 = _0.0436 mM^-1^ cm^-1^ = molar extinction coefficient of hydrogen peroxide; C_prot_ = final concentration of the shoot protein extract)

For superoxide dismutase (SOD) activity, 1 mL of the reaction mixture contained 50 mM potassium phosphate buffer (pH 7.8), 10 mM L-methionine, 57 µM nitroblue tetrazolium (NBT) chloride, 100 µM EDTA, 2 µM riboflavin, and a dilution series of the shoot protein extracts. The reaction mixture was incubated in the presence of a 15 W white fluorescent light for 10 minutes, in a closed cardboard box tightly wrapped in a black cotton cloth, at 25°C. Spectrophotometric measurements at 540 nm were performed over the dilution series to quantify one unit (U) of SOD activity as the concentration of tissue extract necessary to achieve 50% inhibition of NBT photoreduction.

For each genotype, the activities of POD, CAT, and SOD were calculated relative to the mean values measured at the 0 mM treatment, similar to the calculation of relative plant height, relative shoot fresh weight, relative water saturation deficit, or relative chlorophyll content.

### 2.5 Data analysis

SAS software (SAS Institute, 2002, SAS/STAT 9.00, SAS Institute Inc., Cary, NC, USA) was used for statistical analysis. Data points represent the mean values ± standard errors (SE) of at least 3 (for water saturation deficit, chlorophyll content, SOD, POD, and CAT activity) or at least 30 biological replicates (for salinity injury index, plant height, and shoot fresh weight). Additionally, the frequency values (for rooting and acclimatization efficiency) represent the means (± SE) of at least 3 replicates of experiments, each consisting of at least 30 biological replicates unless otherwise stated. The mean values of the measured parameters were compared through one-way analysis of variance (ANOVA), and statistical reliability of the differences between the mean values was determined by Fisher’s least significant differences (LSD) *post-hoc* test (*P*< 0.05). The results were visualized in OriginPro 8.5 (OriginLab Corporation, Northampton, MA, USA).

For principal component analysis (PCA), the FactoMineR package (Lê et al., 2008) was run in R version 4.2.2 (R Core Team, 2022). This package was also used for the construction of vector plots of variables, whereas the package Factoextra (Kassambara and Mundt, 2020) was used for graphical representation of data clustering.

## 3 Results

### 3.1 Macroscopic salinity injury and overall viability

**Figure 1:**
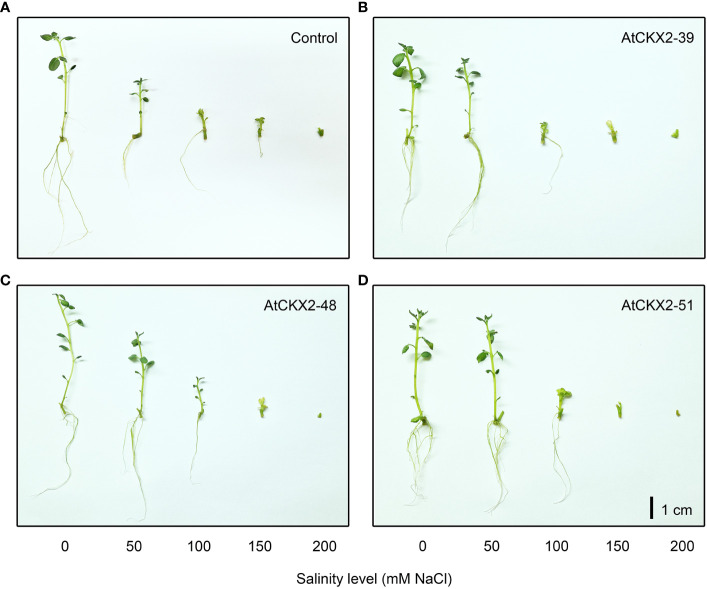
Typical appearance of non-transformed control **(A)**, *AtCKX2*-39 **(B)**, *AtCKX2*-48 **(C)**, and *AtCKX2*-51 **(D)** plants after 20 days of exposure to 0 mM, 50 mM, 100 mM, 150 mM, and 200 mM NaCl (from left to right) in *in vitro* culture. Size bar = 1 cm.

**Figure 2:**
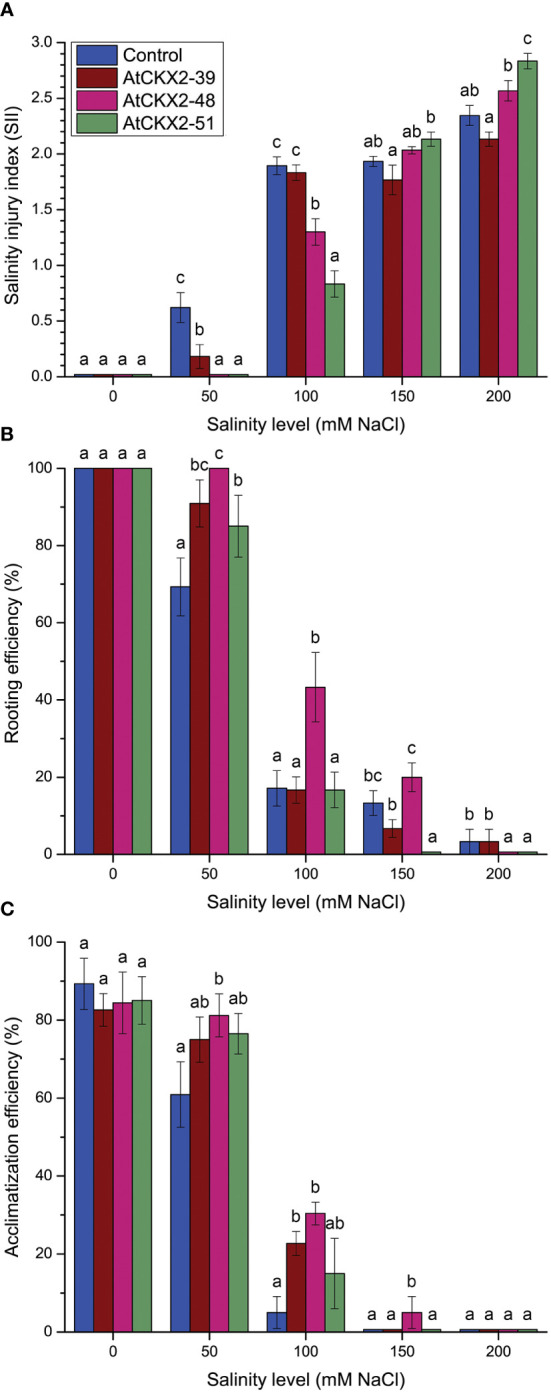
Parameters of salinity injury and overall viability of non-transformed control and *AtCKX2*-transgenic potato plants after 20 days of exposure to 0 mM, 50 mM, 100 mM, 150 mM, and 200 mM NaCl in *in vitro* culture: salinity injury index (SII) **(A)**, rooting efficiency **(B)**, and acclimatization efficiency **(C)**. Data represent mean values ± standard errors (*n* ≥ 30). Within each experimental treatment, the mean values marked with different letters and without a letter in common are statistically different from each other (*P*< 0.05 according to the Fisher’s LSD *post-hoc* test).

The *in vitro* grown plantlets of all the four potato lines showed considerable sensitivity to the salinity treatments, which progressively increased from mild (50 mM) to severe (200 mM) NaCl levels (**Figure 1**). Macroscopic evaluation of the salinity injury revealed that the values of the salinity injury index (SII) progressed from 0 to 3 along with the increasing salinity (**Figure 2A**). In all the potato lines, SII was scored as 0 in all plants at the 0 mM NaCl treatment. However, differences in SII values were observed between the control and *AtCKX2*-transgenic potato lines when salinity was applied. At 50 mM NaCl, the control plants responded with an increase in the mean SII value (0.62), whereas the mean SII value of the *AtCKX2*-39 plants was much lower (0.18), and the plants of the other two transgenic lines appeared not to suffer any visible injury. At 100 mM NaCl, the *AtCKX2*-39 plants responded similarly to the control plants (mean SII = 1.8-1.9), whereas the other two transgenic lines showed some degree of salinity injury (0.8-1.3), albeit significantly lesser compared to control or *AtCKX2*-39 plants. The increasing effects of salinity injury at higher concentrations of NaCl appeared to progress more quickly in *AtCKX2*-48 and *AtCKX2*-51 plants compared to control and *AtCKX2*-39. Thus, at 150 mM NaCl, all four lines showed similar levels of salinity injury, with no significant differences between genotypes (1.7-2.1). Furthermore, at 200 mM NaCl the *AtCKX2*-51 plants were significantly more sensitive than control plants (**Figure 2A**) and all died before the end of the experiment.

Rooting efficiency was measured to quantify the deleterious effects of salinity on root development of the control and *AtCKX2*-transgenic potato plants (**Figure 2B**). All plants of all the four genotypes rooted regularly on MS media, with 100% efficiency at 0 mM NaCl. At 50 mM NaCl, the rooting efficiency of the non-transformed control dropped to 69%, whereas the values remained significantly higher for all three *AtCKX2*-transgenic lines; in *AtCKX2*-48 plants, the rooting efficiency was still 100%, as was at the 0 mM NaCl treatment. However, the rooting efficiency declined sharply in all four potato genotypes at 100 mM NaCl. For *AtCKX2*-39, *AtCKX2*-51 and the non-transformed control, rooting efficiency was around 17% with almost identical values; for *AtCKX2*-48, it was significantly higher (43%). At 150 mM NaCl, *AtCKX2*-39 and *AtCKX2*-48 did not significantly differ from the non-transformed control, but rooting was completely lost in *AtCKX2*-51. At 200 mM NaCl, rooting was lost in both *AtCKX2*-48 and *AtCKX2*-51, whereas in both control and *AtCKX2*-39 plants, the appearance of a single, stunted and necrotic root was observed in about 3% of the plants (**Figure 2B**).

To examine the differences in recovery potential after salt stress in different genotypes, rooted plants from different salinity treatments were acclimatized in soil pots and their survival after 7 days was quantified. Similar acclimatization efficiency, ranging from 85% to 90%, was observed for 0 mM NaCl-grown plants, with no significant differences between genotypes (**Figure 2C**). All potato lines except *AtCKX2*-48 showed reduced acclimatization efficiency after exposure to 50 mM NaCl, compared to the 0 mM treatment. *AtCKX2*-48 showed significantly increased acclimatization compared to the non-transformed control upon 50 mM NaCl treatment. After exposure to 100 mM NaCl, the acclimatization efficiency decreased sharply for all genotypes, but the values for *AtCKX2*-39 and *AtCKX2*-48 remained higher than those for the non-transformed control. Furthermore, a single *AtCKX2*-48 plant survived 7 days of acclimatization after the 150 mM treatment, out of three trials comprising 6-7 rooted plants each (**Figure 2C**).

### 3.2 Parameters of shoot growth

**Figure 3:**
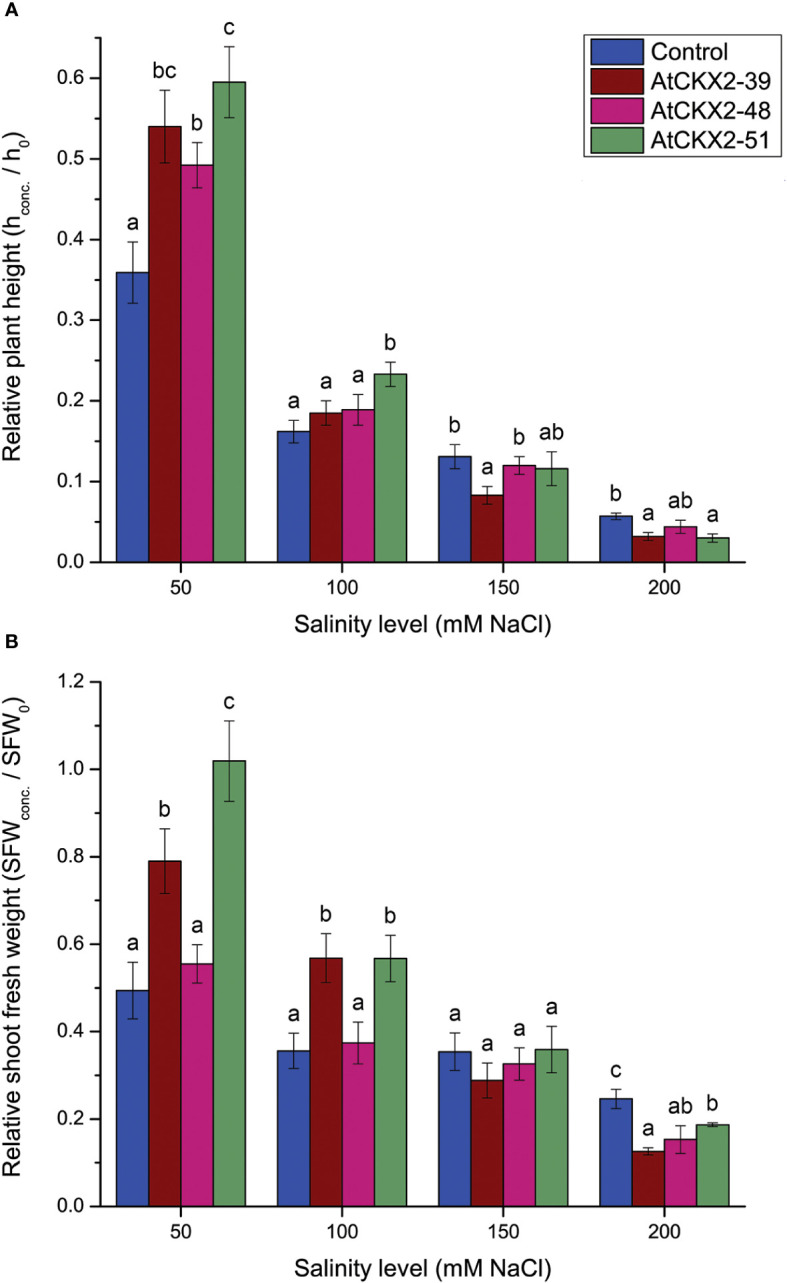
Parameters of shoot growth: relative plant height **(A)** and relative shoot fresh weight (SFW) **(B)** calculated for the non-transformed control and *AtCKX2*-transgenic potato plants after 20 days of exposure to 50 mM, 100 mM, 150 mM, and 200 mM NaCl in *in vitro* culture. The plant height and SFW values are calculated as fold-change compared to 0 mM NaCl for each genotype. Data represent mean values ± standard errors (*n* ≥ 30). Within each experimental treatment, the mean values marked with different letters and without a letter in common are statistically different from each other (*P*< 0.05 according to the Fisher’s LSD *post-hoc* test).

Next, we quantified the shoot growth responses in the control and *AtCKX2*-transgenic potato lines to different salinity levels by plotting the effects of salinity on plant height and shoot fresh weight (**Figure 3**). Since the four potato lines differed in mean shoot length under control conditions due to the effects of the CK deficiency on shoot growth (Raspor et al., 2012), we assumed that comparing absolute plant height or absolute shoot fresh weight at the different salinity levels would not provide a realistic picture of the specific effects of salinity on shoot growth. A more accurate approximation of the specific effects of salinity on shoot growth of each genotype can be calculated by expressing the values for plant height and shoot fresh weight relative to the mean plant height (or mean shoot fresh weight) of the respective genotype at 0 mM NaCl. Therefore, the values of relative plant height and shoot fresh weight are shown in **Figure 3** instead of the absolute values. The absolute values for plant height and shoot fresh weight at the 0 mM treatment are shown in the **Supplementary Figure 1**.

Similar to the SII values, measurements of relative plant height showed milder responses of *AtCKX2*-transgenic lines to lower salinity levels, whereas their responses to higher salinity levels were more pronounced than those of the control plants (**Figure 3A**). At 50 mM NaCl, the mean relative shoot length of the control plants was about 0.36, whereas plant height was significantly less affected in the *AtCKX2*-transgenic potato lines, decreasing to 0.5-0.6 of their respective 0 mM NaCl mean values. At 100 mM NaCl, plant height decreased to 0.16-0.19 of the 0 mM NaCl average value in all potato lines except *AtCKX2*-51, in which the mean relative plant height was significantly greater (0.23) than in the non-transformed control line. At 150 mM NaCl, relative plant height was significantly lower (0.08) in *AtCKX2*-39 than in the non-transformed control line, whereas the other potato lines had values ranging from 0.11 to 0.13. Finally, at 200 mM NaCl, the non-transformed control line showed significantly greater relative plant height (0.057) compared to *AtCKX2*-39 and *AtCKX2*-51 (**Figure 3A**).

As with SII measurements and relative plant height, the response of relative shoot fresh weight (SFW) of the *AtCKX2*-transgenic potato lines to salinity was generally milder than in the non-transformed control at the lower levels of salinity, whereas it gradually increased and became more pronounced than in the control plants at higher salinity levels (**Figure 3B**). At 50 mM NaCl, the *AtCKX2*-48 plants showed a pattern similar to that of control plants, whereas the mean relative SFW of *AtCKX2*-39 and *AtCKX2*-51 plants remained significantly higher than that of control plants. Notably, the mean relative SFW of *AtCKX2*-51 plants was close to 1.0 (1.02 ± 0.09), indicating that their SFW remained unaffected compared to the 0 mM NaCl conditions. At 100 mM, the relative SFW dropped in all the four potato lines, but remained significantly higher in *AtCKX2*-39 and *AtCKX2*-51 compared to the non-transformed control. However, at 150 mM NaCl, the mean relative SFW dropped to about 0.3 in all the potato lines with no significant differences between genotypes, and furthermore, at 200 mM NaCl, all the *AtCKX2*-transgenic lines were significantly more affected than the non-transformed control, with the relative SFW values ranging from 0.12 to 0.19 (**Figure 3B**).

### 3.3 Salinity-induced physiological damage

**Figure 4:**
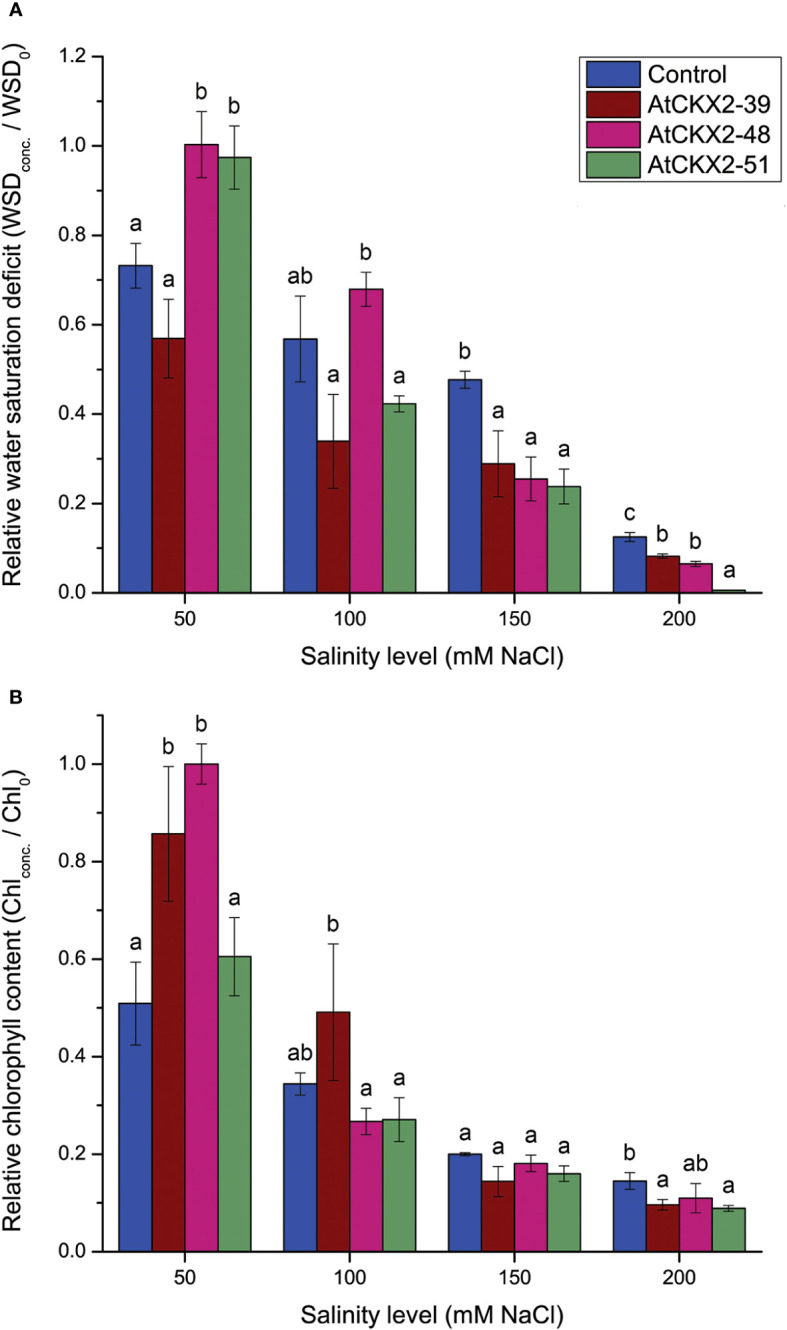
Parameters of damage to homeostasis: relative water saturation deficit (WSD) **(A)** and relative chlorophyll content **(B)** in the leaves of the non-transformed control and *AtCKX2*-transgenic potato plants after 20 days of exposure to 50 mM, 100 mM, 150 mM, and 200 mM NaCl in *in vitro* culture. The WSD and chlorophyll content are calculated as fold-change compared to 0 mM NaCl for each genotype. Data represent mean values ± standard errors (*n* = 3). Within each experimental treatment, the mean values marked with different letters and without a letter in common are statistically different from each other (*P*< 0.05 according to the Fisher’s LSD *post-hoc* test).

Salinity stress-induced physiological damage was assessed by measuring water saturation deficit (WSD) and leaf chlorophyll (Chl) content (**Figure 4**). To compensate for the effects of the genotype-dependent differences that are unrelated to salinity, we expressed WSD and the chlorophyll content as relative values for each genotype, related to their respective mean values at 0 mM NaCl. The absolute values of WSD and chlorophyll content at 0 mM NaCl are shown in **Supplementary Figure 1**.

Water saturation deficit (WSD) was determined as a measure of loss of osmotic balance in salinity-stressed plants (**Figure 4A**). As salinity increased, the mean WSD values progressively dropped to near zero for all the potato lines in the 200 mM treatment (**Figure 4A**). At the 50 mM treatment, the mean relative values for *AtCKX2*-48 and *AtCKX2*-51 were close to 1, indicating that the water saturation of the leaves of these two genotypes remained unchanged compared to the 0 mM treatment. In contrast, the mean relative WSD for control plants was significantly lower (0.73), indicating a change in water uptake in control plants already at 50 mM NaCl. Furthermore, at 100 mM NaCl, the mean relative WSD values ranged from 0.34 in *AtCKX2*-39 to 0.68 in *AtCKX2*-48, but without significant differences compared to the non-transformed control line (0.57). However, the decrease in the mean relative WSD values of the *AtCKX2*-transgenic lines progressed more rapidly at higher NaCl concentrations, thus all the three *AtCKX2* lines had significantly lower values than the non-transformed control at both 150 and 200 mM NaCl, with *AtCKX2*-51 reaching 0 at the highest NaCl concentration (**Figure 4A**).

The relative chlorophyll content was determined in the leaves of the four potato lines to assess the oxidative damage caused by their exposure to the different salinity levels (**Figure 4B**). At 50 mM NaCl, the total chlorophyll content in the leaves of non-transformed control plants already decreased to half of that measured at 0 mM NaCl (relative value = 0.51), whereas significantly higher relative chlorophyll content was recorded in *AtCKX2*-39 and *AtCKX2*-48. For *AtCKX2*-48, the mean total chlorophyll content recorded at 50 mM NaCl equaled the mean value measured at 0 mM NaCl (relative value = 1.00). However, chlorophyll content decreased significantly in all four potato lines at 100 mM NaCl, and decreased further at 150 mM NaCl, with no significant differences between the non-transformed control and any of the transgenic lines. Finally, at 200 mM NaCl, the relative chlorophyll content dropped to 0.145 for the non-transformed control, whereas the relative values for the *AtCKX2*-transgenic lines ranged from 0.09 to 0.11, with *AtCKX2*-39 and *AtCKX2*-51 having significantly lower values than the non-transformed control (**Figure 4B**).

### 3.4 Antioxidant enzymes

**Figure 5:**
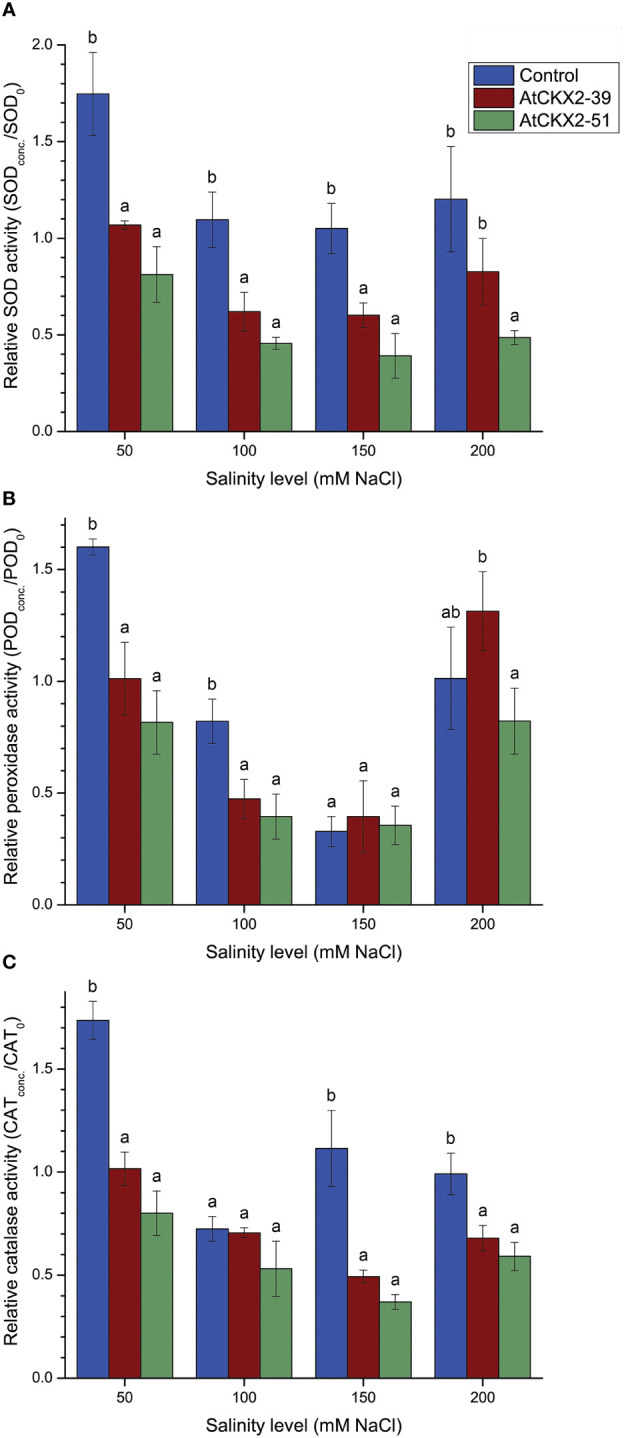
Parameters of enzymatic antioxidant response: relative superoxide dismutase (SOD) **(A)**, peroxidase (POD) **(B)**, and catalase (CAT) **(C)** activity in the shoots of the non-transformed control, *AtCKX2*-39, and *AtCKX2*-51 plants after 20 days of exposure to 50 mM, 100 mM, 150 mM, and 200 mM NaCl in *in vitro* culture. The relative enzyme activities are calculated as fold-change compared to 0 mM NaCl for each genotype. Data represent mean values ± standard errors (*n* = 3). Within each experimental treatment, the mean values marked with different letters and without a letter in common are statistically different from each other (*P*< 0.05 according to the Fisher’s LSD *post-hoc* test).

The ability of non-transformed and *AtCKX2*-transgenic potato plants to mitigate the oxidative damage associated with salinity stress was assessed by measuring the total activity of superoxide dismutase (SOD), peroxidase (POD), and catalase (CAT) in shoots of potato plants exposed to different salinity levels. Since all these three enzymes showed considerable genotype-dependent variations at 0 mM NaCl (**Supplementary Figure 1**), their values were normalized relative to the corresponding values at 0 mM NaCl, as previously explained (**Figure 5**). No significant difference was observed between the shoots of control and *AtCKX2*-48 plants in the activity of any of the measured antioxidant enzymes in any of the treatments (data not shown). In contrast, the profiles of relative SOD, POD, and CAT activity in the shoots of *AtCKX2*-39 and *AtCKX2*-51 plants were significantly different from control plants, suggesting that the CK deficiency generally weakens the response of antioxidant enzymes to salinity in these two CK-deficient potato lines (**Figure 5**).

The profile of the antioxidant response of total superoxide dismutases (SOD) to salt stress is shown in **Figure 5A**. At 50 mM NaCl, the activity of SOD increased by approximately 75% in the shoots of non-transformed control compared to SOD activity at 0 mM NaCl (relative value = 1.75). It was significantly lower in *AtCKX2*-39 and *AtCKX2*-51, where it remained unchanged or even decreased compared to 0 mM NaCl. At 100 mM NaCl, the relative SOD activity decreased in all the potato lines, but remained significantly higher in the non-transformed control than in the lines *AtCKX2*-39 and *AtCKX2*-51. At 150 mM and 200 mM NaCl, the relative SOD activity maintained similar values to 100 mM NaCl in each genotype and remained significantly lower in *AtCKX2*-39 and *AtCKX2*-51 compared to the non-transformed control (**Figure 5A**).

The profile of the antioxidant response of total peroxidases (POD) to salt stress is shown in **Figure 5B**. At 50 mM NaCl, the activity of POD increased by approximately 60% in the shoots of the non-transformed control compared to 0 mM NaCl (relative value = 1.60). In *AtCKX2*-39 shoots, the POD activity remained virtually unchanged compared to 0 mM NaCl (relative value = 1.01), whereas it decreased in *AtCKX2*-51 (relative value = 0.82). Briefly, at 50 mM, the relative POD activity was significantly higher in non-transformed control than in *AtCKX2*-39 and *AtCKX2*-51. This relationship was maintained at 100 mM NaCl, although the POD activity was decreased in all the potato lines compared to 50 mM NaCl (**Figure 5B**). At 150 mM NaCl, the POD activity continued to decrease in all the potato lines compared to 100 mM NaCl, with relative POD values ranging from 0.33 to 0.39, but without significant differences between the potato lines. Interestingly, POD activity increased sharply in all the potato lines at 200 mM NaCl, with relative POD values now ranging from 0.82 to 1.31, but still with no statistically significant differences from the non-transformed control (**Figure 5B**).

The profile of total catalases (CAT) in response to salt stress (**Figure 5C**) showed certain general similarities with the profile of SOD and POD. At 50 mM NaCl, the CAT activity in different potato lines showed a profile similar to that of both SOD and POD enzymes: the total activity of CAT increased compared to 0 mM NaCl in the non-transformed control, remained similar to 0 mM NaCl in *AtCKX2*-39, and reached a relative value below 1 in *AtCKX2*-51. Similar to SOD and POD, the relative CAT values at 50 mM NaCl were lower for the transgenic lines compared to the non-transformed control. At 100 mM NaCl, the relative CAT activity decreased for all potato lines, with no significant differences between the genotypes. At higher salinity levels (150 mM and 200 mM), relative CAT values remained close to 1 or slightly higher for the non-transformed control plants, whereas they were significantly lower for *AtCKX2*-39 and *AtCKX2*-51 (**Figure 5C**).

### 3.5 Principal component analysis

**Figure 6:**
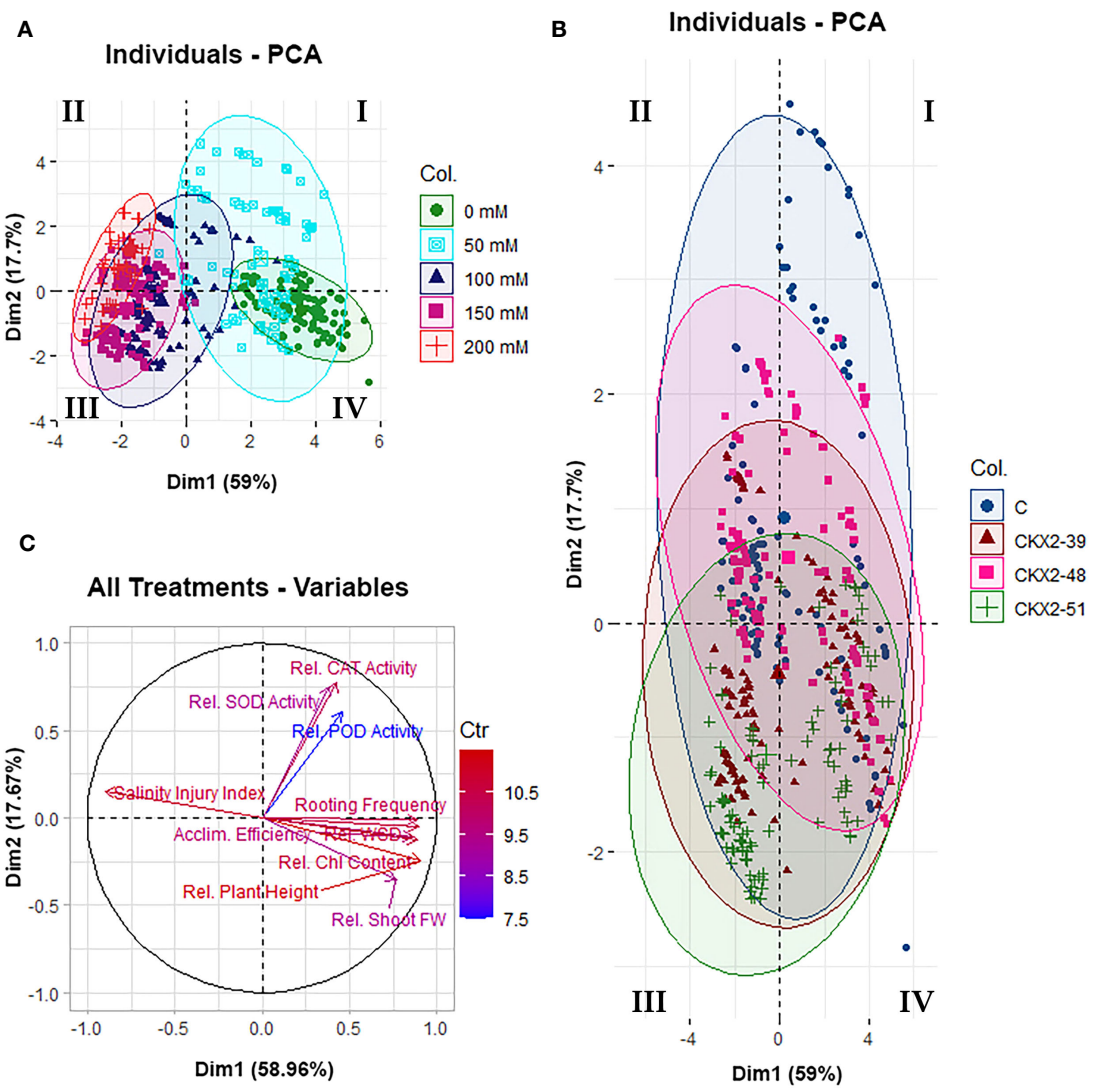
Principal component analysis (PCA) of all data from all the five experimental treatments (0 mM, 50 mM, 100 mM, 150 mM, and 200 mM NaCl) applied in this study: data clustering for different experimental treatments **(A)**, data clustering for different potato genotypes **(B)**, and vector plot of variables from all the five treatments **(C)**. The diagrams were constructed using the packages Factoextra **(A, B)** and FactoMineR **(C)** in R. The Roman numerals (I, II, III, IV) in the data clustering diagrams **(A, B)** indicate the quadrants of the Cartesian coordinate system, as per mathematical convention.

**Figure 7:**
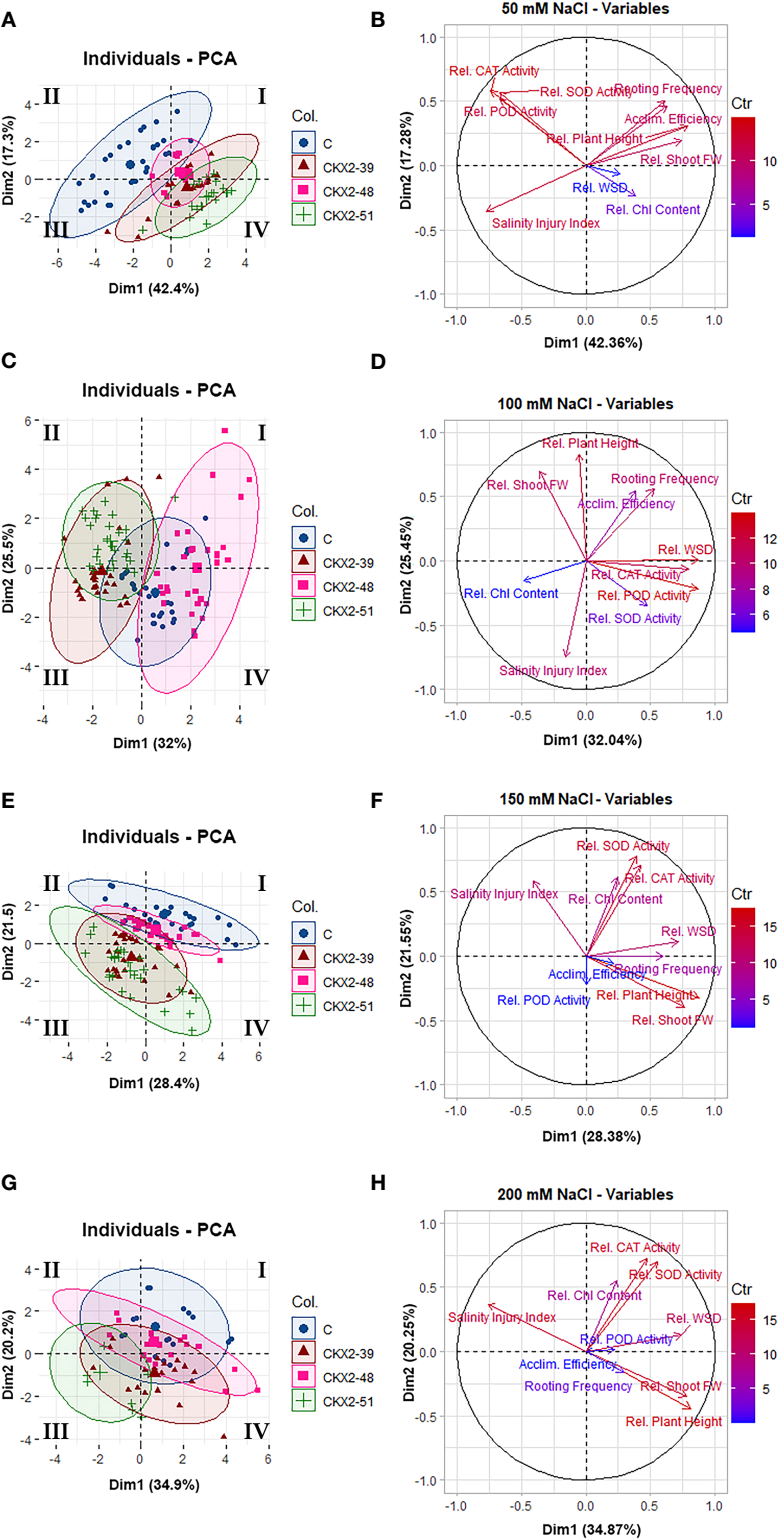
Principal component analysis (PCA) of data from individual salinity treatments: 50 mM NaCl **(A, B)**, 100 mM **(C, D)**, 150 mM **(E, F)**, and 200 mM **(G, H)** NaCl. The data clustering diagrams given on the left **(A, C, E, G)** were constructed using the package Factoextra in R. The Roman numerals (I, II, III, IV) in the data clustering diagrams indicate the quadrants of the Cartesian coordinate system, as per mathematical convention. The vector plots of variables shown on the right **(B, D, F, H)** were constructed using the package FactoMineR in R.

To gain a deeper understanding of the general relations between the responses of the four potato genotypes to the different levels of salt stress, we performed a principal component analysis (PCA) of the parameters quantified in this study (**Figures 6**, **7**).

**Figure 6** summarizes the results of PCA performed on the total pool of data of all experiments (all salinity levels from 0 mM to 200 mM NaCl) for all the four genotypes. The analysis shows that the two principal components of variability account for 59.0% and 17.7% of the total data variability, respectively (**Figure 6**). The distribution of data collected from the different salt treatments shows that the different salinity levels are roughly aligned along the x-axis corresponding to the principal component 1 (PC1), progressively decreasing from 200 mM to 0 mM NaCl from the negative (left-hand side) to the positive (right-hand side) end of the x-axis (**Figure 6A**). When the total collected data are broken down by different potato genotypes, a serial distribution of the data along the y-axis is observed, roughly corresponding to PC2. Although the distribution of the data corresponding to the different potato genotypes shows considerable overlaps between the data domains corresponding to particular genotypes, a shift along the y-axis is observed, with the data corresponding to the non-transformed control shifted towards the upper (positive) end of the y-axis, followed by data corresponding to *AtCKX2*-48 and *AtCKX2*-39, whereas the data corresponding to *AtCKX2*-51 are shifted toward the bottom (negative) end of the y-axis (**Figure 6B**). In addition, the vector plot of variables from all treatments in the experiment shows considerable alignment of variables closely associated with salt stress (rooting frequency, water saturation deficit, acclimatization efficiency, chlorophyll content, salinity injury index), with the x-axis corresponding to PC1 (**Figure 6C**). As expected, the vector representing the salinity injury index is directed towards the negative end of the x-axis (**Figure 6C**), consistent with SII reaching higher values in the plants cultured at higher salt concentrations, for which the data are clustered to the negative end of the x-axis (**Figure 6A**). The remaining variables aligned with the x-axis are directed toward its positive end (**Figure 6C**), consistently with reaching higher values at low salinity treatments, for which the data domains are clustered toward the positive end of the x-axis (**Figure 6A**). The vectors corresponding to plant height and shoot fresh weight show less collinearity with PC1, whereas SOD, POD and CAT activity appear to cluster very closely to each other, but cannot be associated with either PC1 or PC2 (**Figure 6C**).

**Figure 7** shows separate principal component analyses performed for each of the four salinity treatments (50, 100, 150, and 200 mM). Since each graph corresponds to data from a single salinity treatment, the salinity levels do not contribute to the variability of the data within each individual graph; therefore, the contribution of PC1 to data variability within each plot is less dominant than the contribution of PC1 in **Figure 6**, and here it is related to factors other than salinity. Overall, less clear correlations can be made between the principal components of data variability and most of the measured parameters-variables in the individual salinity treatments (**Figures 7B, D, F, H**). However, useful insights can be obtained when the data plots are broken down to individual genotypes (**Figures 7A, C, E, G**).

At 50 mM NaCl, there is relatively little overlap between the data domain of the non-transformed control on the one hand, and the data domains of the *AtCKX2*-transgenic lines on the other, with the non-transformed control data shifted to the quadrant II of the PCA matrix (**Figure 7A**). The data for *AtCKX2*-39, and especially for *AtCKX2*-51, are shifted in the opposite direction (**Figure 7A**). On the vector plot of variables, this observation can be matched with the orientation of the vectors corresponding to SOD, POD, and CAT on the one hand (consistent with the higher relative values of these parameters in the non-transformed control at 50 mM NaCl) and the opposite orientation of the Chl vector on the other hand (consistent with higher relative Chl content in *AtCKX2*-transgenic plants in this treatment) (**Figure 7B**).

At 100 mM NaCl, the most striking feature is the relative repositioning of the data domains corresponding to the different potato genotypes, resulting in the central positioning of the non-transformed control, whereas the data domain corresponding to *AtCKX2*-48 is positioned opposite the other two *AtCKX2*-transgenic lines (**Figure 7C**). On the variables plot, the orientation of the Chl vector coincides with the distribution of data for *AtCKX2*-39, whereas the data domain corresponding to *AtCKX2*-48 coincides with the orientation of the vector for the water saturation deficit (**Figure 7D**).

Further repositioning of the data domains occurs at 150 mM NaCl, resulting again in a shift of the data domain corresponding to the non-transformed control toward the positive end of the y-axis at the top of the PCA matrix. In addition, the data domains corresponding to all four genotypes show a tendency to converge toward the quadrant II, while becoming more divergent in the direction of the quadrant IV (**Figure 7E**). On the variables plot, the orientation of the salinity injury vector is directed to the part of the PCA matrix where the data domains of the four genotypes converge (**Figure 7F**). This area of the matrix corresponds to higher values of salinity injury, i.e., plants of different genotypes all responding to high salinity by sustaining high salinity damage, resulting in similar patterns of data distribution across all genotypes. The opposite orientation of vectors associated with the features of viability such as “rooting frequency”, “plant height”, and “shoot fresh weight” (**Figure 7F**), indicates that the plants that were still viable at 150 mM NaCl tended to display more pronounced genotype-dependent differences in data patterns, leading to more pronounced divergence between data domains corresponding to different genotypes in the right-hand part of the PCA matrix (**Figure 7E**).

At 200 mM NaCl, the data domain of the non-transformed control plants remained shifted to the upper part of the PCA matrix, whereas the data domain of *AtCKX2*-51 shifted to the quadrant III (**Figure 7G**). On the vector plot of variables, the vector corresponding to salinity injury is oriented to point to the negative end of the x-axis (**Figure 7H**), which is consistent with the data domain corresponding to *AtCKX2*-51, the only genotype that showed no survival at this NaCl concentration (**Figure 7G**). The vectors corresponding to plant height and shoot fresh weight are anti-collinear with salinity injury (**Figure 7H**), whereas the orientation of the SOD, CAT, and Chl vectors (**Figure 7H**) more or less coincides with the position of the data domain of the non-transformed control in the PCA matrix (**Figure 7G**).

## 4 Discussion

Exposure to salinity induces physiological and physical damage in plants through the toxic effects of Na^+^ and Cl^-^ ions and induction of oxidative stress (Munns and Tester, 2008). Exposure to a range of NaCl concentrations in the nutrient media resulted in visible changes in *in vitro* grown potato plants, that could be readily correlated with the applied salinity levels (**Figure 1**). Comparison between the potato plants exposed to 50, 100, 150, and 200 mM NaCl reveals a general trend of increasing visible salinity injury, decreasing rooting and viability (**Figure 2**), a decrease in shoot growth (**Figure 3**), and increasing damage to osmotic homeostasis and chlorophyll integrity (**Figure 4**) along with the increase in applied salinity levels. These trends are consistent with the extensive current knowledge on the toxic effects of salinity and associated oxidative stress on photosynthetic tissue integrity and consequent chlorophyll degradation and shoot growth interruption, as well as root tissue damage and associated root growth arrest (Verslues et al., 2006; Munns and Tester, 2008; Flowers et al., 2015). In addition, the profiles of SOD, POD, and CAT activity in the shoots of stressed potato plants showed somewhat more complex patterns, with a more or less consistent decrease in enzyme activity from 50 mM to 150 mM NaCl, followed by a sharp increase in POD activity at 200 mM NaCl in all potato lines (**Figure 5B**), and stagnation, or less pronounced increase in SOD and CAT activity (**Figure 5A, C**).

Principal component analysis (PCA) of the total pool of data from all the experiments in this work revealed that the two principal components, accounting for 59% and 18% of the total data variability, can be roughly associated with the data variability among the treatments and among the genotypes examined in this study, respectively (**Figure 6A, B**). This observation raises an important implication: although data variability was primarily influenced (59%) by the different salinity levels to which the plants were exposed, an additional component of data variability, accounting for 18% of total data variability, was largely genotype-dependent. Furthermore, genotype-dependent clustering of data along the y-axis of the PCA plot (control > *AtCKX2*-48 > *AtCKX2*-39 > *AtCKX2*-51) suggests that the three *AtCKX2*-transgenic lines showed a common tendency to differentiate from the control genotype, with *AtCKX2*-51 showing the greatest divergence from the non-transformed control (**Figure 6B**). Even when the transgenic lines are derived from the same genotype before transformation and contain the same transgene, they differ among themselves at the genotypic and phenotypic levels, which is a consequence of position effect (van Leeuwen et al., 2001) and epigenetic silencing (Matzke and Matzke, 1998). Accordingly, *AtCKX2*-51 was shown to have the highest activity of the transgenic CKX enzyme in both shoot and root tissues, and to be more phenotypically distinct from the non-transformed control in terms of morphological and other traits compared to *AtCKX2*-39 and *AtCKX2*-48 (Raspor et al., 2012). Thus, the greatest divergence of *AtCKX2*-51 from the general data patterns of the control plants in this study, according to PCA, is consistent with our previous knowledge of the general characteristics of this transgenic line.

The observed differences between the control and *AtCKX2*-transgenic potato lines could not be generalized as greater or lesser salt tolerance of one or the other potato genotype. Rather, the results of this study can be summarized as follows: the *AtCKX2*-transgenic potato lines showed greater tolerance to lower salinity levels compared to the non-transformed control, but became more sensitive at higher salinity levels due to CK deficiency. For instance, the mean salinity injury index of the control plants at 50 mM NaCl was significantly higher than that of any of the *AtCKX2*-transgenic lines, whereas at 200 mM NaCl, it was significantly lower than that of *AtCKX2*-51 (**Figure 2A**). Accordingly, at least two of the *AtCKX2*-transgenic lines performed significantly better than control plants in terms of rooting efficiency, plant height and shoot fresh weight at 50 mM NaCl, but significantly weaker than control plants when cultured at 200 mM NaCl (**Figures 2**, **3**). Similar relations were observed for water saturation deficit and chlorophyll content (**Figure 4**).

For *AtCKX2*-48 and *AtCKX2*-51, several parameters, indicative of salt stress, showed relative values around 1.0 when measured at 50 mM NaCl, indicating that their absolute values remained unchanged compared to the 0 mM NaCl treatment. Accordingly, the salinity injury index of all the plants of these two genotypes equaled 0 at 50 mM NaCl (**Figure 2A**), indicating that no macroscopic signs of salinity-induced damage were observed. Moreover, rooting efficiency, water saturation deficit, and chlorophyll content remained unchanged in *AtCKX2*-48 at 50 mM NaCl compared to the 0 mM NaCl treatment, as did water saturation deficit and shoot fresh weight in *AtCKX2*-51 (**Figures 2**-**4**). In addition, the activities of antioxidant enzymes were not up-regulated (relative values ≈ 1) in the shoots of *AtCKX2*-39 and *AtCKX2*-51 at 50 mM compared to 0 mM NaCl (**Figure 5**). Conversely, all of these parameters were strongly affected in the non-transformed control plants, showing a 30-60% increase or decrease compared to 0 mM NaCl, as shown by their relative values at 50 mM NaCl (**Figures 2**-**5**). Thus, the 50 mM NaCl concentration, which already significantly affected the performance of the non-transformed potato plants, had no (or only a very weak) stressful effect on the *AtCKX2*-transgenic plants. This generalization is corroborated by PCA performed on the data obtained at this NaCl concentration. Here, there is an obvious differential clustering of the *AtCKX2*-transgenic genotypes, especially *AtCKX2*-51, away from the center of variation of the data domain corresponding to the control genotype (**Figure 7A**).

At 100 mM NaCl, the measured parameters continue to indicate either a greater tolerance of the *AtCKX2*-transgenic lines compared to control, or a lack of obvious difference in tolerance (**Figures 2**-**4**). At 150 mM NaCl, the differences between the control and the *AtCKX2*-transgenic lines are lost for most of the measured parameters, with the exception of the water saturation deficit, which already shows a reversal of the trend that was evident at lower salinity levels (**Figure 4A**), and of SOD and CAT activity, with the antioxidant SOD and CAT responses being severely impaired in *AtCKX2*-39 and *AtCKX2*-51 compared to control (**Figure 5A, C**). At 200 mM NaCl, none of the measured parameters indicate a better tolerance of the *AtCKX2*-transgenic lines compared to the control line; in fact, many of them indicate the opposite (**Figures 2**-**5**) which is also corroborated by the PCA results (**Figure 7G, H**).

Previous research has demonstrated a role for the negative regulation of salt tolerance by the CK signaling system in *Arabidopsis* (Nishiyama et al., 2011; Nishiyama et al., 2012; Li et al., 2019; Abdelrahman et al., 2021). Thus, CK-deficient *Arabidopsis* plants overexpressing homologous *CKX* genes (Nishiyama et al., 2011), or heterologous *CKX* genes from *Medicago sativa* (Li et al., 2019), as well as multiple-order mutant lines deficient in CK biosynthesis (Nishiyama et al., 2011; Nishiyama et al., 2012) or signaling (Abdelrahman et al., 2021) showed enhanced tolerance to salinity, even up to 200 mM NaCl. It has been suggested that the higher tolerance of CK signaling-deficient mutants to salt stress is related to metabolomic changes, including enhanced accumulation of primary and secondary metabolites that contribute to the mitigation of osmotic and oxidative stress associated with salinity (Abdelrahman et al., 2021). Improved salt tolerance of an *AtCKX2*-transgenic line was also reported in centaury (*Centaurium erythraea* Rafn.) (Trifunović-Momčilov et al., 2020). In Chinese cabbage (*Brassica rapa* subsp. *pekinensis*), the *BrCKX* genes, coding for enzymes involved in CK catabolism, were found to contain regulatory elements responsive to ABA, enabling their transcription under abiotic stresses such as drought or salinity, and enhancing CK degradation (Liu et al., 2013). This regulatory circuit enables a shift in the balance between CK as a stress response-delaying phytohormone and ABA as a positive regulator of the stress response (Chele et al., 2021). Taken together, these reports suggest that down-regulation of CK signaling is generally beneficial to the plant responses to salt stress.

In the *AtCKX2*-transgenic lines used in this study, the endogenous levels of bioactive CK forms are reduced to about 25% of the bioactive CK pool of normal potato plants (Raspor et al., 2012). Thus, our findings on the enhanced tolerance of *AtCKX2*-transgenic potato lines to low (50 mM) and intermediate (100 mM) NaCl concentrations are consistent with the existing knowledge on the roles of CK deficiency in tolerance to salinity. However, the reversal of this expected relationship around 150 mM NaCl and the subsequent increased sensitivity of *AtCKX2*-transgenic potato lines at 200 mM NaCl suggest that in potato, the “price” of CK deficiency appears to outweigh its relative benefits when the salinity level exceeds 150 mM NaCl. Most previous reports on the relationship between CK deficiency and salt tolerance concern research performed on *Arabidopsis*, with the tolerance of CK-deficient genotypes being maintained even at the highest NaCl concentrations (Nishiyama et al., 2011; Nishiyama et al., 2012; Li et al., 2019; Abdelrahman et al., 2021). Although the beneficial effects of CK deficiency on the tolerance to mild salinity likely apply to all flowering plants, species-specific differences, e.g., between potato and *Arabidopsis*, could appear at higher NaCl concentrations, likely due to differences in the regulation of particular stress tolerance mechanisms at higher salinity levels.

Plants possess a wide variety of abiotic stress tolerance mechanisms, not all of which are recruited immediately upon exposure to stress. The recruitment of some of them is rather dependent on the duration and severity of stress. Moreover, differences in the efficiency of certain stress tolerance mechanisms may occur not only among species, but also among genotypes within a single species (Munns and Tester, 2008; Napar et al., 2022). For instance, early activation of antioxidant enzymes may be considered indicative of an efficient stress response, whereas their late activation in response to salinity is more likely to be observed in sensitive genotypes (Munns and Tester, 2008). In our study, SOD, POD, and CAT were readily up-regulated in the non-transformed control but not in *AtCKX2*-39 and *AtCKX2*-51 at 50 mM NaCl, and were even down-regulated in the two transgenic genotypes at 100 mM and 150 mM NaCl (**Figure 5**). The activity of POD decreased in all potato lines at 100 mM NaCl and dropped further at 150 mM but increased again significantly at 200 mM, indicating a final up-regulation of peroxidases in the dying plants at the highest NaCl concentration (**Figure 5B**). Compared with POD, the profiles of SOD and CAT activity showed less variation across the range of NaCl concentrations, but were characterized by notably lower relative activity in both *AtCKX2*-39 and *AtCKX2*-51 compared to control plants almost over the entire range of NaCl concentrations (**Figure 5A, C**). Thus, the initial lack of up-regulation of antioxidant enzymes in the two *AtCKX2*-transgenic lines at 50 mM NaCl can be interpreted in two ways, which are not necessarily mutually exclusive. First, the antioxidant enzymes are not up-regulated in the *AtCKX2*-transgenic plants because at 50 mM NaCl, these plants are not experiencing stress; or alternatively, a normal mechanism of timely up-regulation of antioxidant enzymes in response to mild salt stress, which functions in non-transformed control plants, appeared to be impaired in the *AtCKX2*-39 and *AtCKX2*-51 plants. This might have ultimately contributed to the poor performance of these two transgenic lines at higher NaCl concentrations.

Previous research has shown that exogenous application of CK enhances the antioxidant response of SOD, POD, and CAT to salt stress in eggplant (Wu et al., 2012), perennial ryegrass (Ma et al., 2016), and faba bean (Abdel Latef et al., 2021). In potato, a recent genome-wide characterization of *StSOD* genes revealed that their promoters contain regulatory elements responsive to various phytohormones, although none of them appear to be directly regulated by CK (Rudić et al., 2022). Thus, it is conceivable that the impairment of up-regulation of antioxidant enzymes in response to salinity, observed in *AtCKX2*-39 and *AtCKX2*-51 in our work, was either a direct or indirect consequence of CK deficiency. This impairment of the enzymatic component of the antioxidant response is at least partly responsible for the poor tolerance of *AtCKX2*-transgenic lines to higher salt concentrations; however, since non-enzymatic antioxidant mechanisms such as osmolyte accumulation were not examined in our study, additional impairment of these mechanisms as a consequence of CK deficiency cannot be excluded.

Overall, our results suggest that CK deficiency may have contrasting effects on the tolerance to mild or severe salinity in *in vitro* grown potato. Cytokinin plays roles in a wide range of processes related to plant growth, development, and environmental responses, and interacts extensively with other phytohormones and endogenous signals, hence CK deficiency may have pleiotropic effects, especially on physiological processes in which other regulatory pathways (in addition to CK) are significantly involved (Raspor et al., 2021). Thus, the reasons for impaired tolerance of CK-deficient potato to elevated salinity might be different from those underlying its superior tolerance to mild salinity. Hence, unlike in *Arabidopsis* or centaury, in potato the “price” of CK deficiency appears to outweigh its relative benefits when salinity exceeds 150 mM NaCl. Importantly, these conclusions are drawn from experiments performed *in vitro* and should be only taken as indicative of how field-grown plants might respond to salinity, as it has been repeatedly shown that the behavior of field-grown potato plants may deviate from the patterns observed *in vitro*, due to complex interactions between potato genotypes and the *ex vitro* environment (Cingel et al., 2015; Raspor et al., 2020).

## 5 Conclusions

Evaluation of the responses of cytokinin-deficient *AtCKX2*-transgenic potato lines to different salinity levels was carried out *in vitro*. Morphometric measurements as well as the assessment of parameters of water balance, oxidative damage, and enzymatic stress response, revealed a complex pattern of potato tolerance to salinity. Thus, cytokinin-deficient potato genotypes were significantly more tolerant to low levels of salinity than the non-transformed control plants, but the difference in stress tolerance between genotypes decreased at intermediate salinity, whereas the *AtCKX2*-transgenic lines showed weaker tolerance to high NaCl concentrations than the control plants. Our results challenge the widely accepted assumption that cytokinin deficiency is generally beneficial to salt tolerance. Instead, we propose that cytokinin may play multiple roles in salt tolerance, with cytokinin deficiency worsening rather than alleviating the effects of high NaCl concentrations in a species-specific fashion.

## Data availability statement

The original contributions presented in the study are included in the article/**Supplementary Material**. Further inquiries can be directed to the corresponding author.

## Author contributions

MR: Conceptualization, Data curation, Investigation, Methodology, Resources, Supervision, Validation, Writing – review & editing. MM: Data curation, Formal analysis, Investigation, Visualization, Writing – original draft. JS: Conceptualization, Methodology, Resources, Supervision, Validation, Writing – review & editing. TĆ: Validation, Visualization, Writing – review & editing. ARK: Formal analysis, Visualization, Writing – original draft. NP: Investigation, Writing – original draft. AC: Investigation, Validation, Writing – review & editing. NG: Investigation, Writing – review & editing. VM: Funding acquisition, Validation, Writing – review & editing. SN: Conceptualization, Funding acquisition, Project administration, Resources, Validation, Writing – review & editing.

## References

[B1] Abdel Latef A. A. H. Akter A. Tahjib-Ul-Arif M. (2021). Foliar application of auxin or cytokinin can confer salinity stress tolerance in *Vicia faba* L. Agronomy 11, 790. doi: 10.3390/agronomy11040790

[B2] Abdelrahman M. Nishiyama R. Tran C. D. Kusano M. Nakabayashi R. Okazaki Y.. (2021). Defective cytokinin signaling reprograms lipid and flavonoid gene-to-metabolite networks to mitigate high salinity in *Arabidopsis* . Proc. Natl. Acad. Sci. U.S.A. 118, e2105021118. doi: 10.1073/pnas.2105021118 34815339 PMC8640937

[B3] Ahmad P. Jaleel C. A. Salem M. A. Nabi G. Sharma S. (2010). Roles of enzymatic and nonenzymatic antioxidants in plants during abiotic stress. Crit. Rev. Biotechnol. 30, 161–175. doi: 10.3109/07388550903524243 20214435

[B4] Ahmadu T. Abdullahi A. Ahmad K. (2021). “The role of crop protection in sustainable potato (*Solanum tuberosum* L.) production to alleviate global starvation problem: An overview,” in Solanum tuberosum - A Promising Crop for Starvation Problem. Eds. Yildiz M. Ozgen Y. (London, UK: IntechOpen Limited), 19–51. doi: 10.5772/intechopen.100058

[B5] Barrs H. D. (1968). “Determination of water deficit in plant tissues,” in Water Deficits and Plant Growth, vol. 1 . Ed. Kozlowski T. T. (Clayton, Australia: CSIRO Publishing), 235–368.

[B6] Barrs H. D. Weatherley P. E. (1962). A re-examination of the relative turgidity technique for estimating water deficits in leaves. Austral. J. Biol. Sci. 15, 413–428. doi: 10.1071/BI9620413

[B7] Bradford M. M. (1976). A rapid and sensitive method for the quantitation of microgram quantities of protein utilizing the principle of protein-dye binding. Anal. Biochem. 72, 248–254. doi: 10.1016/0003-2697(76)90527-3 942051

[B8] Campos J. F. Cara B. Pérez-Martín F. Pineda B. Egea I. Flores F. B.. (2016). The tomato mutant *ars1* (*altered response to salt stress 1*) identifies an R1-type MYB transcription factor involved in stomatal closure under salt acclimation. Plant Biotechnol. J. 14, 1345–1356. doi: 10.1111/pbi.12498 26578112 PMC11388943

[B9] Chele K. H. Tinte M. M. Piater L. A. Dubery I. A. Tugizimana F. (2021). Soil salinity, a serious environmental issue and plant responses: A metabolomics perspective. Metabolites 11, 724. doi: 10.3390/metabo11110724 34822381 PMC8620211

[B10] Chourasia K. N. Lal M. K. Tiwari R. K. Dev D. Kardile H. B. Patil V. U.. (2021). Salinity stress in potato: Understanding physiological, biochemical and molecular responses. Life 11, 545. doi: 10.3390/life11060545 34200706 PMC8228783

[B11] Cingel A. Savić J. Ćosić T. Raspor M. Ghalawenji N. Smigocki A.. (2015). Phenotypic performance of transgenic potato (*Solanum tuberosum* L.) plants with pyramided rice cystatin genes (*OCI* and *OCII*). Arch. Biol. Sci. 67, 957–964. doi: 10.2298/ABS141201058C

[B12] Ciura J. Kruk J. (2018). Phytohormones as targets for improving plant productivity and stress tolerance. J. Plant Physiol. 229, 32–40. doi: 10.1016/j.jplph.2018.06.013 30031159

[B13] Diaz-Vivancos P. Faize M. Barba-Espin G. Faize L. Petri C. Hernández J. A.. (2013). Ectopic expression of cytosolic superoxide dismutase and ascorbate peroxidase leads to salt stress tolerance in transgenic plums. Plant Biotechnol. J. 11, 976–985. doi: 10.1111/pbi.12090 23750614

[B14] Flowers T. J. Munns R. Colmer T. D. (2015). Sodium chloride toxicity and the cellular basis of salt tolerance in halophytes. Ann. Bot. 115, 419–431. doi: 10.1093/aob/mcu217 25466549 PMC4332607

[B15] Hanin M. Ebel C. Ngom M. Laplaze L. Masmoudi K. (2016). New insights on plant salt tolerance mechanisms and their potential use for breeding. Front. Plant Sci. 7 1787. doi: 10.3389/fpls.2016.01787 27965692 PMC5126725

[B16] Hartmann A. Senning M. Hedden P. Sonnewald U. Sonnewald S. (2011). Reactivation of meristem activity and sprout growth in potato tubers require both cytokinin and gibberellin. Plant Physiol. 155, 776–796. doi: 10.1104/pp.110.168252 21163959 PMC3032466

[B17] Inskeep W. P. Bloom P. R. (1985). Extinction coefficients of chlorophyll a and *b* in *N,N*-dimethylformamide and 80% acetone. Plant Physiol. 77, 483–485. doi: 10.1104/pp.77.2.483 16664080 PMC1064541

[B18] Joshi R. Sahoo K. K. Tripathi A. K. Kumar R. Gupta B. K. Pareek A.. (2018). Knockdown of an inflorescence meristem-specific cytokinin oxidase - OsCKX2 in rice reduces yield penalty under salinity stress condition. Plant Cell Environ. 41, 936–946. doi: 10.1111/pce.12947 28337744

[B19] Jung C. Seo J. S. Han S. W. Koo Y. J. Kim C. H. Song S. I.. (2008). Overexpression of *AtMYB44* enhances stomatal closure to conifer abiotic stress tolerance in transgenic *Arabidopsis* . Plant Physiol. 146, 623–635. doi: 10.1104/pp.107.110981 18162593 PMC2245844

[B20] Kassambara A. Mundt F. (2020). “Factoextra: Extract and visualize the results of multivariate data analyses,” in R Package Version 1.0.7. Vienna, Austria: R Foundation for Statistical Computing. Available at: https://CRAN.R-project.org/package=factoextra.

[B21] Kolachevskaya O. O. Myakushina Y. A. Getman I. A. Lomin S. N. Deyneko I. V. Deigraf S. V.. (2021). Hormonal regulation and crosstalk of auxin/cytokinin signaling pathways in potatoes in *vitro* and in relation to vegetation or tuberization stages. Int. J. Mol. Sci. 22, 8207. doi: 10.3390/ijms22158207 34360972 PMC8347663

[B22] Kronzucker H. J. Britto D. T. (2011). Sodium transport in plants: A critical review. New Phytol. 189, 54–81. doi: 10.1111/j.1469-8137.2010.03540.x 21118256

[B23] Lê S. Josse J. Husson F. (2008). FactoMineR: An R package for multivariate analysis. J. Stat. Software 25, 1. doi: 10.18637/jss.v025.i01

[B24] Li S. An Y. Hailati S. Zhang J. Cao Y. Liu Y.. (2019). Overexpression of the cytokinin oxidase/dehydrogenase (CKX) from *Medicago sativa* enhanced salt stress tolerance of *Arabidopsis* . J. Plant Biol. 62, 374–386. doi: 10.1007/s12374-019-0141-z

[B25] Linsmaier E. M. Skoog F. (1965). Organic growth factor requirements of tobacco tissue cultures. Physiol. Plantarum 18, 100–127. doi: 10.1111/j.1399-3054.1965.tb06874.x

[B26] Liu Z. Lv Y. Zhang M. Liu Y. Kong L. Zou M.. (2013). Identification, expression, and comparative genomic analysis of the *IPT* and *CKX* gene families in Chinese cabbage (*Brassica rapa* ssp. *pekinensis*). BMC Genomics 14, 594. doi: 10.1186/1471-2164-14-594 24001366 PMC3766048

[B27] Liu M. Pan T. Allakhverdiev S. I. Yu M. Shabala S. (2020). Crop halophytism: An environmentally sustainable solution for global food security. Trends Plant Sci. 25, 630–634. doi: 10.1016/j.tplants.2020.04.008 32444156

[B28] Ma X. Zhang J. Huang B. (2016). Cytokinin-mitigation of salt-induced leaf senescence in perennial ryegrass involving the activation of antioxidant systems and ionic balance. Environ. Exp. Bot. 125, 1–11. doi: 10.1016/j.envexpbot.2016.01.002

[B29] MaChado R. M. A. Serralheiro R. P. (2017). Soil salinity: Effect on vegetable crop growth. Management practices to prevent and mitigate soil salinization. Horticulturae 3, 30. doi: 10.3390/horticulturae3020030

[B30] Matzke M. A. Matzke A. J. M. (1998). Epigenetic silencing of plant transgenes as a consequence of diverse cellular defence responses. Cell. Mol. Life Sci. 54, 94–103. doi: 10.1007/s000180050128 9487390 PMC11147416

[B31] Muhammad I. Shalmani A. Ali M. Yang Q. H. Ahmad H. Li F. B. (2021). Mechanisms regulating the dynamics of photosynthesis under abiotic stresses. Front. Plant Sci. 11. doi: 10.3389/fpls.2020.615942 PMC7876081 33584756

[B32] Munns R. Tester M. (2008). Mechanisms of salinity tolerance. Annu. Rev. Plant Biol. 59, 651–681. doi: 10.1146/annurev.arplant.59.032607.092911 18444910

[B33] Murashige T. Skoog F. (1962). A revised medium for rapid growth and bio assays with tobacco tissue cultures. Physiol. Plantarum 15, 473–497. doi: 10.1111/j.1399-3054.1962.tb08052.x

[B34] Mýtinová Z. Motyka V. Haisel D. Gaudinová A. Lubovská Z. Wilhelmová N. (2010). Effect of abiotic stresses on the activity of antioxidative enzymes and contents of phytohormones in wild type and *AtCKX2* transgenic tobacco plants. Biol. Plantarum 54, 461–470. doi: 10.1007/s10535-010-0082-3

[B35] Napar W. P. F. Kaleri A. R. Ahmed A. Nabi F. Sajid S. Ćosić T.. (2022). The anthocyanin-rich tomato genotype LA-1996 displays superior efficiency of mechanisms of tolerance to salinity and drought. J. Plant Physiol. 271, 153662. doi: 10.1016/j.jplph.2022.153662 35259587

[B36] Nishiyama R. Le D. T. Watanabe Y. Matsui A. Tanaka M. Seki M.. (2012). Transcriptome analyses of a salt-tolerant cytokinin-deficient mutant reveal differential regulation of salt stress response by cytokinin deficiency. PloS One 7, e32124. doi: 10.1371/journal.pone.0032124 22355415 PMC3280229

[B37] Nishiyama R. Watanabe Y. Fujita Y. Le D. T. Kojima M. Werner T.. (2011). Analysis of cytokinin mutants and regulation of cytokinin metabolic genes reveals important regulatory roles of cytokinins in drought, salt and abscisic acid responses, and abscisic acid biosynthesis. Plant Cell 23, 2169–2183. doi: 10.1105/tpc.111.087395 21719693 PMC3160038

[B38] Pardo J. M. Quintero F. J. (2002). Plants and sodium ions: Keeping company with the enemy. Genome Biol. 3, reviews1017. doi: 10.1186/gb-2002-3-6-reviews1017 12093381 PMC139373

[B39] Raspor M. Motyka V. Ninković S. Dobrev P. I. Malbeck J. Ćosić T.. (2020). Endogenous levels of cytokinins, indole-3-acetic acid and abscisic acid in *in vitro* grown potato: A contribution to potato hormonomics. Sci. Rep. 10 3437. doi: 10.1038/s41598-020-60412-9 32103086 PMC7044434

[B40] Raspor M. Motyka V. Ninković S. Malbeck J. Dobrev P. I. Zdravković-Korać S.. (2021). Overexpressing *AtCKX1* in potato plants grown in *vitro*: The effects on cytokinin composition and tuberization. J. Plant Growth Regul. 40, 37–47. doi: 10.1007/s00344-020-10080-w

[B41] Raspor M. Motyka V. Žižková E. Dobrev P. I. Trávníčková A. Zdravković-Korać S.. (2012). Cytokinin profiles of *AtCKX2*-overexpressing potato plants and the impact of altered cytokinin homeostasis on tuberization in *vitro* . J. Plant Growth Regul. 31, 460–470. doi: 10.1007/s00344-011-9255-3

[B42] R Core Team (2022). R: A language and environment for statistical computing (Vienna, Austria: R Foundation for Statistical Computing). Available at: https://www.R-project.org/.

[B43] Romanov G. A. Aksenova N. P. Konstantinova T. N. Golyanovskaya S. A. Kossmann J. Willmitzer L. (2000). Effect of indole-3-acetic acid and kinetin on tuberisation parameters of different cultivars and transgenic lines of potato in *vitro* . Plant Growth Regul. 32, 245–251. doi: 10.1023/A:1010771510526

[B44] Rudić J. Dragićević M. B. Momčilović I. Simonović A. D. Pantelić D. (2022). *In silico* study of superoxidase dismutase gene family in potato and effects of elevated temperature and salicylic acid on gene expression. Antioxidants 11, 488. doi: 10.3390/antiox11030488 35326138 PMC8944489

[B45] Savić J. Nikolić R. Banjac N. Zdravković-Korać S. Stupar S. Cingel A.. (2019). Beneficial implications of sugar beet proteinase inhibitor BvSTI on plant architecture and salt stress tolerance in *Lotus corniculatus* L. J. Plant Physiol. 243, 153055. doi: 10.1016/j.jplph.2019.153055 31639537

[B46] Sharma A. Shahzad B. Kumar V. Kohli S. K. Sidhu G. P. S. Bali A. S.. (2019). Phytohormones regulate accumulation of osmolytes under abiotic stress. Biomolecules 9, 285. doi: 10.3390/biom9070285 31319576 PMC6680914

[B47] Tan C. Kalhoro M. T. Faqir Y. Ma J. Osei M. D. Khaliq G. (2022). Climate-resilient microbial biotechnology: A perspective on sustainable agriculture. Sustainability 14, 5574. doi: 10.3390/su14095574

[B48] Teo H. M. Aziz A. Wahizatul A. A. Bhubalan K. Nordahliawate M. S. S. Syazlie C. I. M.. (2022). Setting a plausible route for saline soil-based crop cultivations by application of beneficial halophyte-associated bacteria: A review. Microorganisms 10, 657. doi: 10.3390/microorganisms10030657 35336232 PMC8953261

[B49] Trifunović-Momčilov M. Motyka V. Dobrev P. I. Marković M. Milošević S. Jevremović S.. (2021). Phytohormone profiles in non-transformed and *AtCKX* transgenic centaury (*Centaurium erythraea* Rafn) shoots and roots in response to salinity stress in *vitro* . Sci. Rep. 11, 21471. doi: 10.1038/s41598-021-00866-7 34728697 PMC8563955

[B50] Trifunović-Momčilov M. Paunović D. Milošević S. Marković M. Jevremović S. Dragićević I.Č.. (2020). Salinity stress response of non-transformed and *AtCKX* transgenic centaury (*Centaurium erythraea* Rafn.) shoots and roots grown in *vitro* . Ann. Appl. Biol. 177, 74–89. doi: 10.1111/aab.12593

[B51] van Leeuwen W. Ruttink T. Borst-Vrenssen A. W. M. van der Plas L. H. W. van der Krol A. R. (2001). Characterization of position-induced spatial and temporal regulation of transgene promoter activity in plants. J. Exp. Bot. 52, 949–959. doi: 10.1093/jexbot/52.358.949 11432912

[B52] Verslues P. E. Agarwal M. Katiyar-Agarwal S. Zhu J. Zhu J. K. (2006). Methods and concepts in quantifying resistance to drought, salt and freezing, abiotic stresses that affect plant water status. Plant J. 45, 523–539. doi: 10.1111/j.1365-313X.2005.02593.x 16441347

[B53] Wu X. Zhu Z. Li X. Zha D. (2012). Effects of cytokinin on photosynthetic gas exchange, chlorophyll fluorescence parameters and antioxidative system in seedlings of eggplant (*Solanum melongena* L.) under salinity stress. Acta Physiol. Plant 34, 2105–2114. doi: 10.1007/s11738-012-1010-2

[B54] Yildiz M. Poyraz İ. Çavdar A. Özgen Y. Beyaz R. (2021). “Plant responses to salt stress,” in Plant Breeding - Current and Future Views. Ed. Abdurakhmonov I. Y. (London, UK: IntechOpen Limited), 143–160. doi: 10.5772/intechopen.93920

[B55] Zhu Y. Jiang X. Zhang J. He Y. Zhu X. Zhou X.. (2020). Silicon confers cucumber resistance to salinity stress through regulation of proline and cytokinins. Plant Physiol. Biochem. 156, 209–220. doi: 10.1016/j.plaphy.2020.09.014 32977177

