# Supplementary material for: Cytokinin deficiency confers enhanced tolerance to mild, but decreased tolerance to severe salinity stress in in vitro grown potato
Source: Front Plant Sci. 2024 Feb 1;14:1296520. doi: 10.3389/fpls.2023.1296520 (PMC10867153; doi:10.3389/fpls.2023.1296520)
Supplement: Supplementary file 1 [file DataSheet_1.docx]

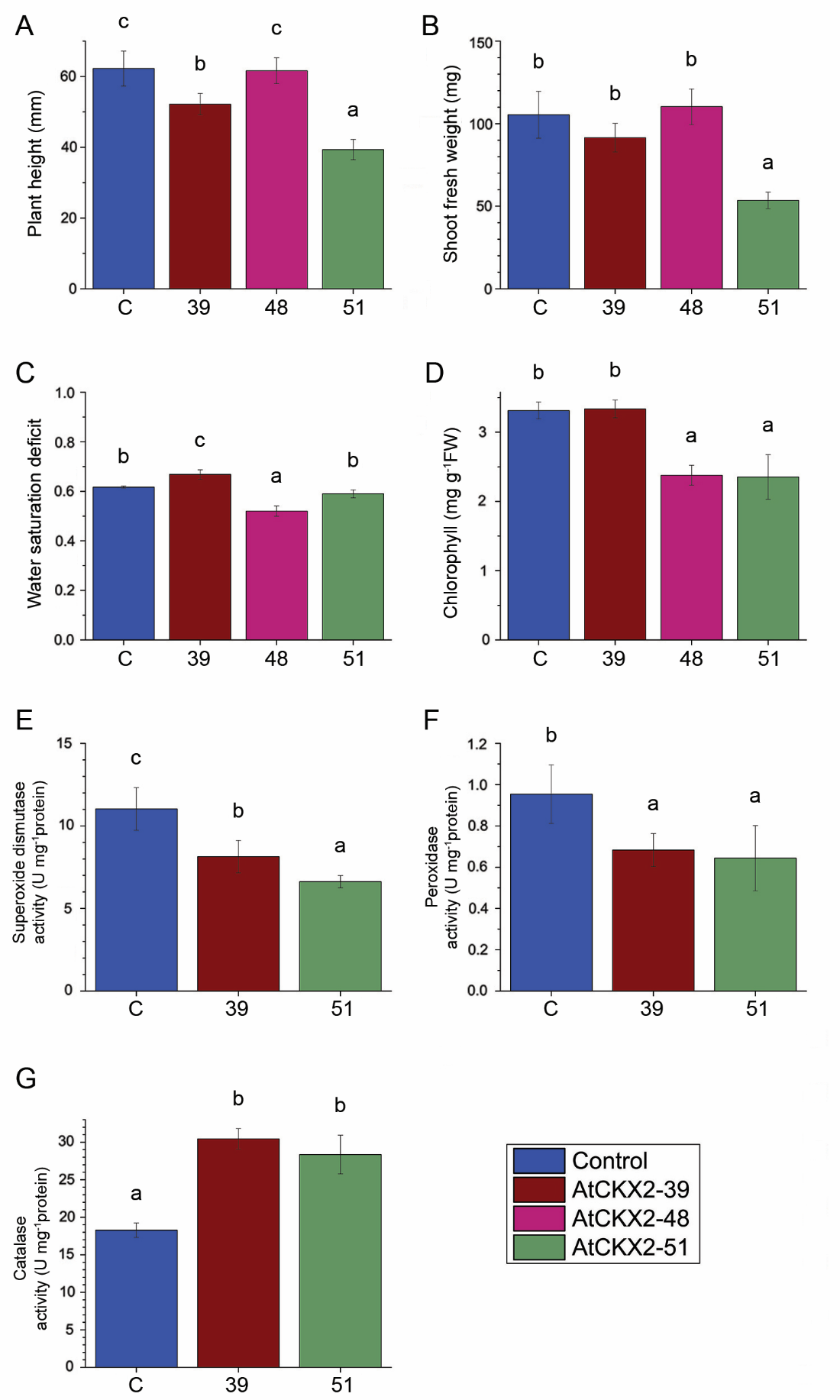


**Supplementary Figure 1.** Genotype-related variations of different parameters measured in 20-day-old *in vitro* grown potato plants at the 0 mM NaCl treatment. Plant height (**A**), shoot fresh weight (**B**), leaf water saturation deficit (**C**), leaf chlorophyll content (**D**), and shoot superoxide dismutase (SOD) (**E**), peroxidase (POD) (**F**), and catalase (CAT) (**G**) activity in non-transformed control ("C"), *AtCKX2*-39 ("39"), *AtCKX2*-48 ("48"), and *AtCKX2*-51 ("51") plants. Data represent mean values ± standard errors (*n* ≥ 30 (**A, B**) or *n* = 3 (**C-G**)). Within each experimental treatment, the mean values marked with different letters are statistically different from each other (*P* < 0.05 according to the Fisher's LSD *post-hoc* test).
